# Reproductive performance, digestibility, and rumen bacteria of goats fed two levels of phytogenic mixture

**DOI:** 10.1186/s13568-025-01961-y

**Published:** 2025-11-03

**Authors:** Alaa Emara Rabee

**Affiliations:** Animal and Poultry Nutrition Department, Desert Research Center, Ministry of Agriculture and Land Reclamation, Cairo, Egypt

**Keywords:** Shami goats, Herbal mixture, Phytochemicals, Rumen bacteria, Feed utilization, Reproduction

## Abstract

Phytogenic mixtures have gathered attention to promote the productive and reproductive efficiency of animals. This study estimated the effect of two levels of phytogenic mixture (HM), including ginger, garlic, artemisia, turmeric, fennel, and fenugreek, on the rumen bacteria and fermentation, digestibility, blood metabolites, and reproductive performance of female Shami goats. Thirty-six female goats were divided into three groups (n = 12) to receive one of three diets: The control diet consisted of alfalfa hay and a concentrate feed mixture (C); control diet contained 1% of the HM based on dry matter intake (H1); control diet contained 2% of the HM (H2). The supplementation modified the rumen bacterial community, which was predominated by phyla Bacteroidota and Firmicutes. The supplementation increased the fibrolytic bacteria, *Prevotella*, *Butyrivibrio*, *Christensenellaceae R-7 group*, *Saccharofermentans*, and *Ruminococcus*. Moreover, the supplementation enhanced the digestibility, total feed intake, total volatile fatty acids (VFA), propionic acid, and iso butyric acid, and reduced the methane production. Additionally, the supplementation enhanced serum glucose, total antioxidant capacity (TAC), and immunoglobulin M (IgM), and decreased cholesterol and urea. The supplementation affected the concentration of serum estrogen and enhanced the reproductive performance indices, including conception and kidding rates. Additionally, higher females were observed in the offspring of group H2. The current herbal mixture can be used at 1% to modify rumen microbiota to enhance digestibility and rumen fermentation as well as reproductive indices.

**Supplementary Information:**

The online version contains supplementary material available at 10.1186/s13568-025-01961-y.

## Introduction

Goats are well-adapted to harsh environments by their grazing habits and physiological characteristics, as they can browse plants that are avoided by other livestock species. Therefore, goats use the natural resources efficiently. Goat productivity in arid regions is constrained by feed shortages and health issues (Rabee et al. 2025a and b). Improving the productivity of goats in the arid areas will contribute to food security with the increase in global warming. Phytogenic feed additives are an emerging solution to improve animal health and productivity, and to be a safe alternative to prohibited antibiotics (Mirzaei et al. 2012). These feed additives are sources of different bioactive compounds such as phenols, tannins, flavonoids, and saponins that improve animal performance through modulating rumen microbial ecosystem and promoting digestibility, rumen fermentation, and feed intake (Mirzaei et al. 2012; Rabee et al. 2024). Furthermore, those compounds improve animal health as they are antipathogens, anti-inflammatories, and immunostimulants (Wang D et al. 2023).

On the other hand, improving the reproductive performance is the main target of the livestock sector (Swelum et al. 2021). Dietary phytochemicals have direct and indirect effects on animal reproduction (Swelum et al. 2021; Wang D et al. 2023). For example, phytochemicals have phytoestrogenic activity as these compounds have estrogen-like chemical structure, which enables them to bind estrogen receptors, causing hormonal disruption and affecting the reproductive performance negatively (Hashem et al. 2024). In contrast, a Chinese herbal mixture was reported to regulate the reproductive hormones, which promoted estrus and reproductive performance through promoting follicle development and ovarian activities (Wang D et al. 2023). Additionally, the phytochemicals have indirect effects on the reproductive performance through improving animal immunity and oxidative status as well as modulating the rumen microbiota, which enhances the feed utilization and the production of VFA and microbial protein (Kholif et al. 2021; Rabee et al. 2024; Wang D et al. 2023).

A herbal mixture of ginger, garlic, artemisia, and turmeric reduced rumen archaea and enhanced the fibrolytic bacteria, including *Prevotella*, *Rikenellaceae RC9 gut group*, and *Ruminococcus*, which reduced methane production and enhanced the digestibility, VFA production, and growth of goats (Rabee et al. 2024, 2025b). Fennel and fenugreek contain galactogogues substances that improve milk production and feed efficiency through improving the synthesis of hormones like prolactin, estrogen, and growth hormone (Penagos Tabares et al. 2014; Abou-Elenin et al. 2016). Similarly, fenugreek seeds supplementation improved feed intake, milk yield, fat-corrected milk, blood prolactin, and triglycerides (Çayiroğlu et al. 2022).

The effect of phytochemicals relies on their types and amounts; in addition, combining different plants in phytogenic mixtures improves the synergistic activities, which improves rumen microbial activities and feed utilization (Kholif et al. 2021; Rabee et al. 2024). Therefore, it was hypothesized that combining fennel and fenugreek into a herbal mixture that was previously used by Rabee et al. (2024) (ginger, garlic, artemisia, and turmeric) could improve the performance of animal production and reproduction. On the other side, the effect of herbal mixtures containing different phytogenic compounds on the reproductive performance in livestock animals is still controversial and needs more studies to clarify the positive and negative effects (Swelum et al. 2021; Hashem et al. 2024; Wang D et al. 2023). Therefore, this study aims to investigate the effect of two levels of herbal mixture (1% and 2% of dry matter feed intake) on the rumen bacteria, digestibility, rumen fermentation, blood metabolites, and reproductive performance of female Shami goats.

## Materials and methods

### Ethics

The study was conducted following the regulations and guidelines of the Institutional Animal Care and Use Committee, Desert Research Center (DRC), Cairo, Egypt (Approval: AN-4-2024). All methods and protocols in this study comply with the ARRIVE 2.0 guidelines and the European animal welfare standards. This study does not include animal slaughtering or clinical trials. All animals were the offspring of the goat herd in Maryout Research Station, DRC, Egypt. At the end of the study, all animals were released to the experimental goat herd.

### Animals and diets

The experiment was carried out at Maryout Research Station, DRC, Alexandria, Egypt. Thirty-six dry female Shami goats (35.85 ± 1.72 kg average body weight; 4–5 years age) were involved in this 120-day experiment. All the animals were the progeny of the goat herd in Maryout Research Station, and were used in the study with permission from the administration of Maryout Research Station and the Animal and Poultry Production Division, DRC. All the animals received the same basal diet that consisted of a 60% concentrate feed mixture (CFM) and 40% Alfalfa hay (*Medicago sativa*) to meet the breeding feeding requirements for goats according to the National Research Council (NRC, 2007). The first group fed on a basal diet without supplementation (C), the second group fed on a basal diet supplemented with a herbal mixture at 1% (H1), following the recommendation of Rabee et al. (2024), and the third group fed on a basal diet supplemented with a herbal mixture at 2% (H2). The herbal mixture contained garlic (*Allium sativum*), artemisia (*Artemisia vulgaris*), ginger (*Zingiber officinale*), turmeric (*Curcuma longa*), Fenugreek (*Trigonella foenum-graecum*), and Fennel (*Foeniculum vulgare*), which were obtained from the commercial market and were mixed in equal quantities (1:1:1:1:1:1). The mixture was mixed with the CFM daily before feeding to confirm full intake. Animals’ weights were recoded every 15 days. Table 1 presents the details of the components and chemical compositions of animal feeds. Orts were weighed, and feed intake was recorded daily. The samples of animal feeds and refused feeds were sampled weekly and dried in a forced-air oven at 65 °C for 48 h. At the end of the experiment, all animals were released to the goat herd without euthanasia.

**Table 1:** The proximate chemical composition and phytochemicals in the animal diets

	CFM*	Herbal mixture	Alfalfa hay
Proximate chemical composition, %			
DM	91.66	90.00	92.00
OM	93.00	89.55	88.00
CP	15.00	12.00	14.00
EE	4.12	5.13	3.23
CF	4.22	12.13	29.43
Ash	7.00	10.40	12.00
Phytochemicals, %			
Phenols %	0.05	0.10	0.09
Flavonoids%	0.40	0.23	0.15
Tannins %	0.17	1.50	0.25
Polyphenols profile, µg/g			
Gallic acid	1.50	19.39	0
Chlorogenic acid	0.10	188.70	0
Caffeic acid	0	61.29	0
Ellagic acid	0	12.12	0.30
Vanillin	0	4.16	0
Rosmarinic acid	0	8.30	0
Resorcinol	0.80	0	0
Catechin	0	1166.82	0
Rutin	0	0	4
Naringenin	0	4.88	0
Daidzein	0	3.64	0
Quercetin	0	0	220.00
Kaempferol	0	0	14.00
Apigenin	0	0	12.00
Phenanthrene	0.40	0	0.30
Pyrocatechol	0.40	0	0
Coumaric acid	0	2.36	5.00
Ferulic acid	0.20	4.72	0.20
Cinnamic acid	2.90	4.90	1.00
Diosmin	0	0	20.00
Quinic	13.70		5.00
Syringic acid	0	3.32	0

^*^Concentrate feed mixture consisted of corn 55%, soybean meal 14%, wheat bran 17%, cotton meal 12%, lime stone 1%, salt 0.75%, Sodium bicarbonate 0.1%, Vitamins and trace minerals 0.25%, Antitoxins 0.1%. DM = Dry matter; OM = Organic matter; CP = Crude protein; EE = Ether extract; CF = Crude fiber

### Digestibility trail

The digestibility trial was conducted 60 days after the start of the study. Goats were adapted to the digestibility cages for seven days before collecting urine and feces for the next seven days. The weights of animals were recorded at the beginning and end of the digestibility trial. Throughout the collecting period, the offered and refused feeds were collected, recorded to determine the feed intake, and representative samples were gathered daily for chemical analysis. Daily feces output was collected, measured, and mixed thoroughly, and a 10% sub-sample of each animal was collected. Subsamples of feces, offered, and refused feeds were pooled into one sample per animal for the whole collection period. The samples were dried at 65 °C for 48 h and ground and stored for the following chemical analysis. Urine was collected daily in jars, acidified using 100 ml of 4N sulphuric acid, quantified, and a 10% sub-sample of each animal was collected to estimate urine nitrogen. Additionally, drinking water intake was recorded daily. The digestibility of the nutrients was quantified using the method of McDonald et al. (2002).

### Rumen sampling and fermentation parameters

Rumen content was collected from the animals three hours after morning feeding, using a stomach tube. The samples were filtered by cheesecloth, and the pH was measured immediately via a pH meter (WPA CD70, ADWA, Szeged, Hungary). The rumen liquids were used to estimate VFA and ammonia (NH_3_-N), as well as microbial DNA isolation. VFA and ammonia were determined as described in Rabee et al. (2025a). Briefly, one mL of rumen liquid was acidified using 0.2 mL meta-phosphoric acid 25%, and then centrifuged at 15,000 rpm for 15 min. The supernatant was used to measure VFA and ammonia. Ammonia was estimated by ammonia assay kits (Biodiagnostic, Cairo, Egypt). VFAs were measured by a gas chromatography system (TRACE 1300, Thermo Fisher Scientific, Waltham, United States) using a capillary column (TR-FFAP 30 m × 0.53 mmL D × 0.5 μm). The nitrogen was used as the carrier gas, and the calibration was conducted using VFAs standards. The methane production was calculated using the equation: Methane yield = 316/propionate + 4.4 (Rabee et al. 2025a).

### Blood samples and serum metabolites analysis

Blood samples were collected from the animals before morning feeding from the jugular vein into Vacutainer tubes. Blood serum was obtained by centrifuging blood samples at 10,000 × g for 5 min. The serum samples were used to determine glucose (GLU, mg/dl), cholesterol, (CHO, mg/dL), triglycerides (TG, mg/dL), albumin (ALB, g/dL), total protein (TP, g/dL), urea (UREA, mg/dL), creatinine, alanine aminotransferase (ALT), aspartate aminotransferase (AST), and total antioxidant capacity (TAC, mmol/L) using commercial kits (Biodiagnostic, Giza, Egypt) following the manufacturer’s protocols. Furthermore, immunoglobulin A, immunoglobulin G, and immunoglobulin M were estimated using enzyme-linked immunosorbent assay (ELISA).

### DNA extraction and PCR amplification

Total microbial DNA was isolated from 500 µL of the rumen sample fluid. The rumen sample was centrifuged at 13,000 rpm for 15 min, and the precipitated pellets were used in DNA isolation using the QIAamp DNA Stool Mini Kit (Qiagen, Hilden, Germany) following the manufacturer’s protocol. DNA concentration and quality were estimated using a Nanodrop spectrophotometer 2000 (Thermo Scientific, Massachusetts, United States) and gel electrophoresis. The rumen bacteria was investigated using the PCR-amplification of the variable V4 region on 16S rDNA using 515F and 926R primer sets. The PCR amplification conditions were: initial denaturation at 94 °C for 3 min; 35 cycles of 94 °C for 45 s, annealing at 50 °C for 60 s, and extension at 72 °C for 90 s; followed by a final extension at 72 °C for 10 min. PCR amplicons were purified and sequenced using the Illumina MiSeq system (Illumina, California, United States).

### Bioinformatics analysis

The bioinformatic analysis was described in Rabee et al. (2025a). Briefly, the generated paired-end raw sequence reads were analyzed through the DADA2 pipeline (version 1.11.3) using the R environment (version 3.5.2) (Callahan et al. 2016). The generated fastq files of sequence reads were demultiplexed, and their quality was checked based on the quality scores. The samples with a quality score > 30 were kept for the following analyses. The sequences were filtered, trimmed, and dereplicated, followed by merging read 1 and read 2 to get denoised sequences. The chimeras were removed from the denoised sequences to generate Amplicon Sequence Variants (ASVs). Taxonomic assignment of ASVs was carried out by assignTaxonomy and assignSpecies functions and was compared using the SILVA reference database (version 138). Alpha diversity indices, including observed ASVs, Chao1, Shannon, and Inverse Simpson, were determined to measure richness and evenness of the bacterial community in the experimental groups. Beta diversity was evaluated using principal coordinate analysis (PCoA) and visualized by phyloseq and ggplot R-packages. The raw sequence data have been deposited in the NCBI sequence read archive (SRA) under accession number: PRJNA1291028.

### Reproduction performance

#### 1-Estrus synchronization and mating

All the animals were free of reproductive disorders and diseases. Estrus was synchronized in the goats of each group (n = 12) with double intramuscular injections of cloprostenol acetate (Stimestrus, 250 µg Cloprostenol/ml, Marcyrl Animal Health, Egypt), 125 μg each, given 11 days (− 11 and 0) apart. Then, the does were presented to fertile bucks fitted with dye markers to detect the does in estrus for 96 h. Estrus detection was performed every 12 h and continued for 4 days after the last PGF2α treatment, after which the bucks were separated from the does and the does were kept under the nutritional treatments for 45 days.

#### 2-Hormonal profiles

Blood samples were collected during the breeding season for 4 days from the jugular vein into vacutainer tubes at zero times (before the second cloprostenol injection) and every 24 h. Blood serum was separated by centrifuging the blood sample at 10,000 × g for 5 min, and then was stored at − 20 °C. The changes in serum estradiol 17-β (E2) profile were measured using competitive solid phase enzyme immunoassay kits (Monobind, USA) according to the manufacturer’s instructions using an ELISA microplate reader (Stat Fax 2000, Awareness Technology, Inc., USA). The intra- and inter-assay CV’s are 9.3 and 9.7% respectively.

#### 3- Reproductive performance indices

Reproductive performance indices in terms of estrus response rate (the number of does in estrus of the total number of treated does), conception rate (the proportion of mated does that conceived), litter size (the number of kids born to does kidded), and sex ratio were recorded for all animals.

### Proximate chemical analysis

Animal feeds, herbal mixture, and fecal samples were analyzed according to the method of AOAC (1997) to estimate dry matter (DM, method 930.15), crude protein (CP, method 954.01), and ether extract (EE, method 920.39), crude fiber (CF, method 978.10). Additionally, urine nitrogen was analyzed according to AOAC (1997).

### Phytochemical compounds in animal feeds

Total flavonoids, total phenols, total tannins, and total saponins were measured in alfalfa hay, CFM, and herbal mixture as described in Rabee et al. (2024). Total phenols were quantified using Folin–Ciocalteu (Kaur and Kapoor 2002). Total tannins was extracted by boiling in water (Balbaa 1986). Total flavonoids were extracted by petroleum ether and 95% ethanol and quantified using the method of Karawaya and Aboutabl (1982). Polyphenol profile was determined by an Agilent 1260 high-performance liquid chromatography (HPLC) (Thermo Scientific, Massachusetts, United States) using a reversed-phase C18 column. The mobile phase contained water (A) and 0.05% trifluoroacetic acid in acetonitrile (B) at a flow rate of 0.9 ml/min (Biswas et al. 2013).

### Statistical analysis

The data of the relative abundances of bacterial phyla and genera were tested for normality and homogeneity using the Shapiro–Wilk test, and non-normal variables were then arcsine transformed. The effect of herbal mixture supplementation level on the differences in feed intake, digestibility of nutrients, rumen fermentation parameters, bacteria, and blood metabolites groups was examined using one-way ANOVA. The effect of supplementation on estrogen levels at different collection times was examined using repeated-measure ANOVA. A post hoc Duncan test was used to determine significant differences at *p* < 0.05. Chi-square of independence was used to compare reproductive performance criteria in terms of kidding rate, litter size, and sex ratio among the experimental groups. Principal component analysis ordination plot (PCA), Pearson correlation analysis, and Permutational multivariate analysis of variance (PERMANOVA) were used to determine the impact of herbal mixture supplementation level on animal performance using the data of feed intake, digestibility of nutrients, rumen fermentation parameters and bacteria, and blood metabolites. The statistical analyses were performed using SPSS v. 20.0 software package (SPSS 1999).

## Results

### Chemical composition

Table 1 presents the chemical composition of the concentrate feed mixture, alfalfa hay, and herbal mixture used in the study. CFM has higher CP and lower CF and ash; alfalfa hay has higher CF and ash; and the herbal mixture has higher EE.

### Phytochemical content in animal diet

The results revealed that the herbal mixture has higher phenols and tannins compared to CFM and AH, while CFM has higher flavonoids (Table 1). Herbal mixture has a diversity of polyphenolic compounds such as gallic acid, chlorogenic acid, caffeic acid, ellagic acid, vanillin, rosmarinic acid, catechin, naringenin, and daidzein (Table 1).

### Feed intake, digestibility of nutrients, and rumen fermentation

Table 2 shows the roughage and total feed intake, digestibility of nutrients, and rumen fermentation parameters in goats supplemented with three levels of herbal mixtures. Group H1 had higher feed intake of DM, OM, CP, and EE than the CC and H2 groups (*p* < 0.05) (Table 2). The supplementation improved the digestibility of nutrients, where the supplemented groups (H1 and H2) showed higher digestibility of OM, EE, and CF (*p* < 0.05) (Table 2). The supplementation affected some of the rumen fermentation parameters. Total VFA showed a numeric increment in supplemented groups (H1 and H2) without a significant difference (*p* > 0.05). Group H1 showed higher propionic, while group H2 showed higher isobutyric (*p* < 0.05). The supplementation decreased the predicted methane production (*p* < 0.05) (Table 2).

**Table 2:** Effect of herbal mixture supplementation level on feed intake, digestibility of nutrients, and rumen fermentation parameters in goats

	CC		H1		H2		Mean	SE	*p*-value
	Mean	SE	Mean	SE	Mean	SE			
Weight	41.30	2.48	32.25	2.70	34.91	2.80	35.85	1.72	0.09
Roughage intake, g/kg^0.75^									
DMI	26.71	0.21	24.35	1.38	22.53	1.89	24.40	0.89	0.17
OMI	24.12	0.19	21.99	1.25	20.34	1.71	22.04	0.80	0.16
CPI	3.56	0.03	3.49	0.14	3.35	0.17	3.46	0.08	0.54
EEI	0.86	0.01	0.80	0.04	0.75	0.06	0.80	0.03	0.20
CFI	7.86	0.06	6.57	0.53	5.80	0.76	6.68	0.37	0.07
Total Intake, g/kg^0.75^									
DMI	54.75^b^	0.51	67.32^a^	1.59	63.43^a^	1.33	62.25	1.46	0.0001
OMI	50.20^c^	0.47	61.94^a^	1.45	58.31^b^	1.21	57.21	1.35	0.0001
CPI	7.76^c^	0.07	9.90^a^	0.18	9.32^b^	0.16	9.07	0.23	0.0001
EEI	2.02^c^	0.02	2.57^a^	0.06	2.40^b^	0.05	2.35	0.06	0.0001
CFI	9.05	0.07	8.60	0.41	8.46	0.31	8.68	0.18	0.44
Digestibility, %									
DMD	69.73	1.94	75.17	1.25	74.77	1.45	73.43	1.02	0.05
OMD	72.38^b^	1.89	77.80^a^	1.10	77.28^a^	1.31	76.03	0.97	0.04
CPD	78.63	1.39	82.06	1.16	81.79	1.23	80.96	0.78	0.15
EED	73.82^b^	1.13	81.32^a^	1.26	82.65^a^	1.27	79.59	1.15	0.0001
CFD	32.57^b^	3.84	42.61^a^	1.25	37.62^ab^	2.35	37.90	1.70	0.048
Nitrogen balance, g N/d	6.43	0.99	7.44	1.18	10.07	1.85	8.07	0.87	0.21
Water intake, ml/d	2711	371	2347	206	2135	374	2379	183	0.47
Rumen fermentation parameters									
PH	6.44	0.03	6.34	0.04	6.35	0.11	6.37	0.04	0.46
Ammonia, mg/dL	11.86	2.53	9.84	1.43	11.04	2.37	10.91	1.18	0.80
VFA	60.28	6.22	70.79	5.23	74.69	5.19	68.72	3.35	0.22
Acetic	40.79	4.17	45.58	4.08	49.37	4.20	45.27	2.40	0.39
Propionic	9.38^b^	1.00	12.90^a^	0.85	12.15^a^	0.81	11.57	0.61	0.04
Iso butyric	1.56^ab^	0.11	1.19^b^	0.19	1.85^a^	0.18	1.52	0.11	0.04
butyric	5.69	0.80	8.39	0.70	8.08	1.48	7.45	0.63	0.16
Iso valeric	1.96	0.26	1.94	0.29	2.31	0.31	2.06	0.16	0.61
valeric	0.90	0.08	0.79	0.09	0.93	0.14	0.87	0.06	0.61
Predicted methane, g /kg DMI	39.87^a^	4.25	29.43^b^	1.64	31.00^b^	2.21	33.18	1.90	0.04

DMI = Dry matter intake; OMI = Organic matter intake; CPI = Crude protein intake; EEI = Ether extract intake; CFI = Crude fiber intake; DMD = Dry matter digestibility; OMD = Organic matter digestibility; CPD = Crude protein digestibility; EED = Ether Extract digestibility; CFD = Crude fiber digestibility; VFA = Volatile fatty acids.^a,b,c,d^ Means within a row with different subscripts differ significantly (*p* < 0.05). SE = Standard error

### Blood metabolites and immunity

The supplementation affected some blood metabolites (Table 3). Blood glucose was significantly higher in H2, followed by H1 and CC groups, respectively (*p* < 0.05). Furthermore, blood cholesterol was significantly decreased in the supplemented groups (H1 and H2) (*p* < 0.05). Blood urea showed its lowest value in group H1 compared to the CC and H2 groups, with a significant difference (*p* < 0.05). TAC and IgM were significantly higher in supplemented groups (H1 and H2) (*p* < 0.05) (Table 3).

**Table 3:** Blood serum metabolites, total antioxidant capacity, and immunity of goat supplemented with different levels of herbal mixture

	CC		H1		H2		Mean	SE	*p*-value
	Mean	SE	Mean	SE	Mean	SE			
GLU (mg/dL)	65.36^b^	0.45	68.49^b^	2.49	79.71^a^	2.99	71.19	2.04	0.002
CHO (mg/dL)	127.17^a^	4.32	79.56^b^	7.43	70.54^b^	6.07	92.43	7.39	0.0001
TG (mg/dL)	47.38	5.87	40.00	2.48	39.36	1.97	42.25	2.27	0.29
TP (g/dL)	7.48	0.21	7.62	0.25	7.45	0.09	7.52	0.10	0.80
ALB (g/dL)	2.99	0.13	2.92	0.14	3.18	0.15	3.04	0.08	0.43
Urea (mg/dL)	50.95^a^	3.78	38.45^b^	4.16	54.55^a^	2.85	47.98	2.68	0.02
ALT (IU/L)	13.97	1.80	16.25	2.38	19.53	2.41	16.58	1.33	0.24
AST (IU/L)	70.21	6.13	56.02	4.33	57.74	4.60	61.32	3.20	0.14
TAC	1.48^b^	0.14	2.17^a^	0.15	2.02^a^	0.13	1.89	0.11	0.01
Creatinine (mg/dL)	1.19	0.15	1.36	0.31	1.99	0.41	1.51	0.19	0.20
IgA	60.06	0.36	59.28	1.62	61.04	0.71	60.13	0.59	0.51
IgG	56.75	3.61	61.43	6.53	62.93	13.81	60.37	4.80	0.88
IgM	12.11^b^	2.53	19.70^a^	1.64	19.57^a^	0.71	17.13	1.42	0.02

GLU = Glucose; CHO = Cholesterol; TG = triglycerides; TP = Total protein; ALB = Albumin; AST = Aspartate aminotransferase; ALT = Alanine aminotransferase; TAC = Total antioxidant capacity; IgA = Immunoglobulin A; IgG = Immunoglobulin G; IgM = Immunoglobulin M.^a,b,c,d^ Means within a row with different subscripts differ significantly (*p* < 0.05). SE = Standard error

### Diversity of rumen bacteria

**Fig. 1:**
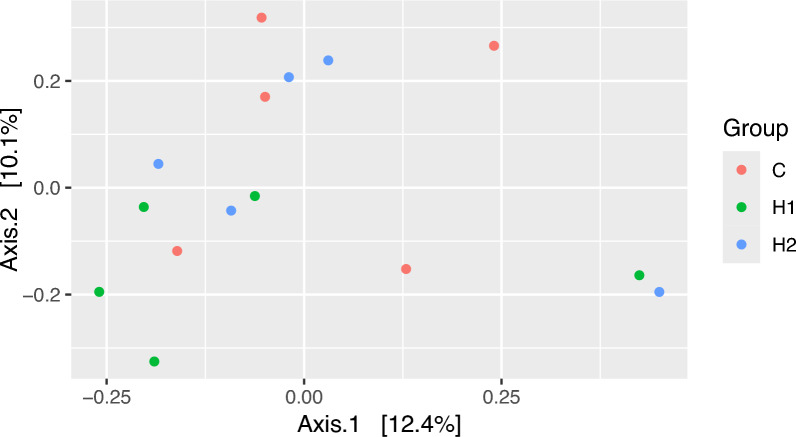
Principal coordinates analysis (PCoA) of the bacterial community was performed based on Bray–Curtis dissimilarity. The analyses were conducted between three goat groups: red circles for the control group (CC), green circles for goats supplemented with 1% herbal mixture (H1), and blue circles for goats supplemented with 2% herbal mixture (H2)

The Illumina sequencing of the V4 region on 16S rDNA amplicons generated a total of 846,452 high-quality sequence reads with an average of 56,430 ± 5503 sequence reads per sample. The supplementation influenced some of the alpha diversity measures significantly (*p* < 0.05) (Table 4) (Supplementary Figure S1). Higher significant values of observed ASVs and Chao1 indices were observed in group H2, followed by groups H1 and CC, respectively (*p* < 0.05). Beta diversity of the rumen bacterial communities of the investigated goats was determined and visualized as principal coordinate analysis (PCoA), which revealed that group H1 was separated from group CC and H2 (Fig. 1).

**Table 4:** Effect of herbal mixture supplementation level on microbial alpha diversity and the relative abundances (%) of bacterial phyla

	CC		H1		H2		Mean	SE	*p*-value
	Mean	SE	Mean	SE	Mean	SE			
Alpha diversity									
Observed ASVs	278.00^b^	34.00	358.00^b^	38.00	469.00^a^	19.00	368.00	26.00	0.004
Chao1	293.00^b^	38.00	389.00^b^	35.00	510.00^a^	25.00	398.00	29.00	0.002
Shannon	3.91	0.30	3.99	0.49	4.50	0.11	4.13	0.19	0.42
Inverse Simpson	24.13	7.47	23.33	8.03	23.81	5.16	23.75	3.74	0.99
Bacterial phyla, %									
Actinobacteriota	0.12	0.07	0.13	0.01	0.23	0.05	0.16	0.03	0.25
Bacteroidota	68.76	2.50	66.40	2.44	57.60	5.22	64.25	2.32	0.11
Cyanobacteria	0.39	0.14	0.15	0.02	0.52	0.14	0.35	0.07	0.11
Firmicutes	16.40^b^	1.60	23.32^b^	0.46	35.73^a^	4.75	25.16	2.64	0.002
Planctomycetota	0.36	0.10	0.33	0.04	0.41	0.01	0.37	0.04	0.63
Proteobacteria	5.62	2.77	7.97	3.35	3.66	0.47	5.75	1.43	0.50
Spirochaetota	7.76^b^	1.30	1.22^a^	0.39	0.81^a^	0.43	3.27	0.96	0.0001
Synergistota	0.08^b^	0.06	0.03^b^	0.01	0.47^a^	0.19	0.19	0.08	0.04
Verrucomicrobiota	0.15	0.01	0.11	0.01	0.08	0.02	0.11	0.01	0.02

ASVs = Amplicon Sequence Variants; ^a,b,c,d^ Means within a row with different subscripts differ significantly (*p* < 0.05). SE = Standard error

### The structure of the bacterial community

Bacterial community was classified into nine bacterial phyla that were dominated by Bacteroidota (64.25%), Firmicutes (25.16%), Proteobacteria (5.75%), and Spirochaetota (3.27%). Bacterial phyla that represented less than 1% of the bacterial community were Actinobacteriota (0.16%), Cyanobacteria (0.35%), Planctomycetota (0.37%), Synergistota (0.19%), and Verrucomicrobiota (0.11%) (Table 4). Phylum Actinobacteriota was not affected by the supplementation (*p *> 0.05). This phylum was dominated by genus *Olsenella*, which was significantly enriched in group H2 (*p* < 0.05) compared to other groups, and genus *Cutibacterium*, which was lower in supplemented groups (*p* < 0.05) (Table 5).

**Table 5:** Effect of herbal mixture supplementation level on the relative abundances (%) of dominant bacterial families and genera

	CC		HM1		HM2		Mean	SE	p-value
	Mean	SE	Mean	SE	Mean	SE			
P: Actinobacteriota									
G: Olsenella	0.04^ab^	0.009	0.035^b^	0.008	0.05^a^	0.01	0.04	0.008	0.02
G: Cutibacterium	0.22^a^	0.05	0.06^b^	0.03	0.03^b^	0.01	0.10	0.03	0.003
P: Bacteroidota									
F: Rikenellaceae; G: Rikenellaceae RC9 gut group	39.62^a^	2.29	36.66^ab^	0.16	31.98^b^	1.81	36.09	1.23	0.02
F: Prevotellaceae	14.55	1.26	20.20	2.26	19.24	1.90	18.00	1.19	0.11
F: Prevotellaceae; G: Prevotella	14.06	1.30	19.81	2.23	18.91	1.86	17.59	1.19	0.045
F: F082	11.81^a^	1.60	7.36^b^	0.32	4.83^b^	1.25	8.01	1.00	0.004
F: Bacteroidales RF16 group	0.64	0.12	0.46	0.01	0.48	0.01	0.53	0.04	0.17
F: Bacteroidales BS11 gut group	1.15^a^	0.16	0.64^b^	0.04	0.40^b^	0.12	0.73	0.10	0.002
F: Muribaculaceae	0.11	0.02	0.49	0.19	0.30	0.05	0.30	0.07	0.09
P: Firmicutes; F: Lachnospiraceae									
F: Lachnospiraceae	1.34^b^	0.23	3.04^b^	0.54	7.37^a^	1.41	3.92	0.83	0.01
G: Butyrivibrio	0.18^b^	0.05	0.64^a^	0.09	0.70^a^	0.04	0.51	0.07	0.0001
G: Lachnospiraceae NK3A20 group	0.09^b^	0.01	0.10^b^	0.03	0.19^a^	0.02	0.13	0.02	0.01
G: Acetitomaculum	0.28^c^	0.05	0.60^b^	0.12	0.98^a^	0.08	0.63	0.09	0.0001
G: Lachnospiraceae XPB1014 group	0.14^b^	0.01	0.10^b^	0.03	2.63^a^	1.01	0.96	0.44	0.01
G: Syntrophococcus	0.05^b^	0.01	0.07^b^	0.01	0.17^a^	0.03	0.10	0.02	0.003
F: Christensenellaceae	1.77^b^	0.32	4.15^b^	0.77	9.93^a^	1.84	5.29	1.11	0.001
G: Christensenellaceae R-7 group	1.44^b^	0.31	3.89^b^	0.71	9.51^a^	1.82	4.95	1.09	0.001
P: Firmicutes; F: Ruminococcaceae									
F: Ruminococcaceae	3.48	0.75	5.83	1.82	3.80	0.38	4.37	0.68	0.32
G: Ruminococcus	0.57	0.13	1.96	0.59	1.35	0.14	1.29	0.24	0.047
G: unclassified_Ruminococcaceae	2.91	0.82	3.87	1.23	2.45	0.24	3.08	0.49	0.51
F: Oscillospiraceae	4.01	0.66	3.49	0.31	5.06	0.43	4.19	0.32	0.11
F: Oscillospiraceae; G: NK4A214 group	1.76^b^	0.33	2.65^b^	0.25	4.09^a^	0.42	2.84	0.31	0.001
F: Hungateiclostridiaceae	0.40^b^	0.06	1.11^b^	0.22	1.29^a^	0.07	0.94	0.13	0.002
F: Hungateiclostridiaceae; G: Saccharofermentans	0.21^b^	0.06	0.84^a^	0.15	0.80^a^	0.11	0.62	0.10	0.003
F: Selenomonadaceae	0.01^b^	0.00	0.02^b^	0.00	0.22^a^	0.08	0.09	0.04	0.011
F: Anaerovoracaceae	0.67^c^	0.06	1.51^b^	0.26	2.38^a^	0.18	1.53	0.21	0.0001
F: Anaerovoracaceae; G: Mogibacterium	0.51^c^	0.04	0.97^b^	0.15	1.39^a^	0.07	0.96	0.11	0.0001
F: Anaerovoracaceae; G: Family XIII AD3011 group	0.07^c^	0.02	0.35^b^	0.09	0.59^a^	0.04	0.34	0.07	0.0001
F: Family XI; G: Anaerococcus	0.14	0.06	0.09	0.00	0.01	0.00	0.08	0.02	0.07
F: Acidaminococcaceae; G: Succiniclasticum	0.06	0.02	0.04	0.00	0.04	0.00	0.05	0.01	0.45
F: Staphylococcaceae; G: Staphylococcus	0.18	0.09	0.17	0.06	0.08	0.00	0.14	0.04	0.45
P: Planctomycetota									
F: Pirellulaceae; G: p-1088-a5 gut group	0.26	0.05	0.17	0.04	0.29	0.02	0.24	0.02	0.13
P: Proteobacteria									
F: Alcaligenaceae	4.21	2.11	6.27	2.65	2.87	0.38	4.45	1.12	0.48
F: Alcaligenaceae; G: Achromobacter	4.13	2.10	5.94	2.51	2.73	0.36	4.27	1.08	0.50
F: Burkholderiaceae	0.61	0.25	0.92	0.35	0.47	0.05	0.67	0.14	0.44
F: Burkholderiaceae; G: Ralstonia	0.45	0.16	0.67	0.24	0.35	0.03	0.49	0.10	0.40
F: Burkholderiaceae; G: Burkholderia-Caballeronia-Paraburkholderia	0.26	0.09	0.22	0.09	0.12	0.02	0.20	0.04	0.45
F: Moraxellaceae; G: Acinetobacter	0.25	0.10	0.17	0.07	0.07	0.01	0.16	0.04	0.27
F: Pseudomonadaceae; G: Pseudomonas	0.08	0.06	0.06	0.03	0.02	0.00	0.05	0.02	0.54
P: Spirochaetota									
F: Spirochaetaceae; G: Sphaerochaeta	7.71^a^	1.31	1.18^b^	0.39	0.77^b^	0.43	3.22	0.96	0.001
P: Synergistota									
F: Synergistaceae; G: Fretibacterium	0.15^ab^	0.09	0.10^b^	0.00	0.50^a^	0.18	0.24	0.08	0.04

P = phylum; F = family; G = genus;^a,b,c,d^ Means within a row with different subscripts differ significantly (*p* < 0.05). SE = Standard error

Phylum Bacteroidota dominated the bacterial community and was not affected by the supplementation. This phylum was dominated by families Rikenellaceae, Prevotellaceae, F082, Bacteroidales RF16, Bacteroidales BS11 gut group, and Muribaculaceae. Family Rikenellaceae was affiliated to genus *Rikenellaceae RC9 gut group* that showed its significantly lower relative abundance in group H2 (*p* < 0.05). Family Prevotellaceae was dominated by genus *Prevotella*, which was significantly higher in the supplemented groups (*p* < 0.05). Families F082 and BS11 gut group were significantly lower in supplemented groups (*p* < 0.05).

Phylum Firmicutes was affiliated with families Lachnospiraceae, Christensenellaceae, Ruminococcaceae, Oscillospiraceae, Hungateiclostridiaceae, Selenomonadaceae, Anaerovoracaceae, Anaerovoracaceae, Anaerovoracaceae; Family XI, Acidaminococcaceae, and Staphylococcaceae (Table 5). Family Lachnospiraceae was affiliated with *Butyrivibrio*, *Lachnospiraceae NK3A20 group*, *Acetitomaculum*, *Lachnospiraceae XPB1014 group*, and *Syntrophococcus,* which were higher in supplemented groups (*p* < 0.05) (Table 5). Family Christensenellaceae was affiliated with genus *Christensenellaceae R-7 group*, which was higher in supplemented groups (H1 and H2) (*p* < 0.05). Family Ruminococcaceae was dominated by genus *Ruminococcus,* which was higher in the supplemented groups (*p* < 0.05) (Table 5). Family Oscillospiraceae was classified as genus *NK4A214 group*, which was enriched in supplemented groups (*p* < 0.05). Family Hungateiclostridiaceae was classified mainly as genus *Saccharofermentans*, which was increased by the supplementation (*p *< 0.05). Family Anaerovoracaceae was classified to genus *Mogibacterium*, which was increased by the supplementation (*p* < 0.05). Family Anaerovoracaceae genus Family XIII AD3011 group, which was higher in supplemented groups (H1 and H2) (*p* < 0.05) (Table 5). Family Anaerovoracaceae was dominated by genus *Anaerovorax*, which was enriched in supplemented groups (H1 and H2) (*p* < 0.05) (Table 5).

Phylum Spirochaetota was classified mainly to the genus *Sphaerochaeta*, which was lower in supplemented groups (H1 and H2) (*p* < 0.05) (Table 5). Moreover, phylum Synergistota was classified mainly to the genus *Fretibacterium*, which showed its higher relative abundance in group H2 (*p* < 0.05) (Table 5).

### Principal component analysis (PCA)and Bray–Curtis permutational multivariate analysis of variance (PERMANOVA)

**Fig. 2:**
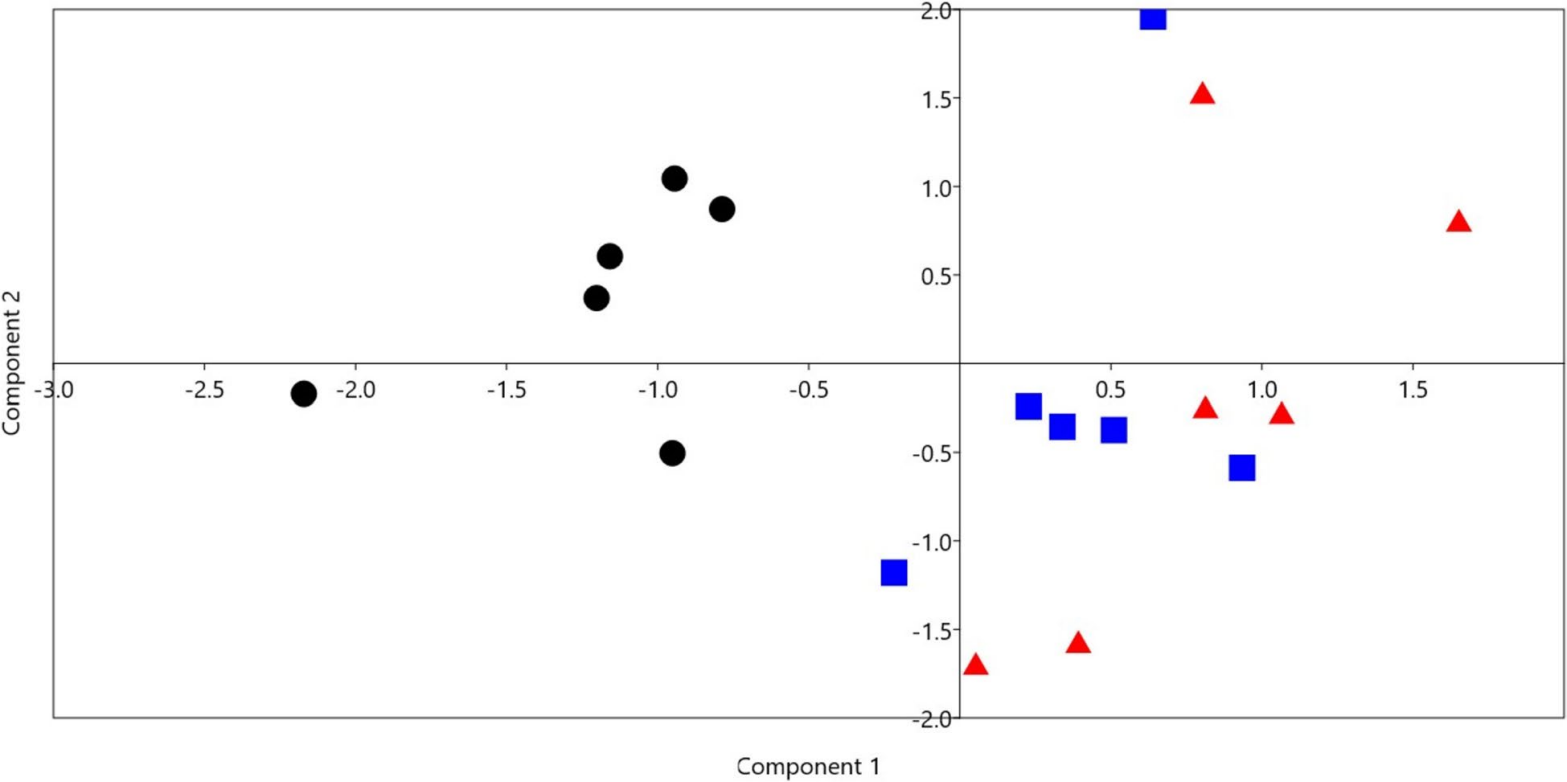
Principal component analysis (PCA) was determined using the results of feed intake, digestibility of nutrients, rumen fermentation parameters and bacteria, and blood metabolites. The black dots are for the control group (CC), the blue squares are for group H1, and the red triangles are for group H2

PCA analysis was performed using data on digestibility, rumen fermentation parameters, blood metabolites, and the relative abundances of dominant bacterial phyla and genera (Fig. 2). The results showed that the samples of the control group were separated from the samples of the supplemented groups (H1, H2). Blood cholesterol, total VFA, IgM, IgG, methane production, and the relative abundance of phylum Firmicutes drove the clustering. The result of PERMANOVA indicated that the difference between the groups was significant (*p* = 0.00001). Pairwise comparison between groups based on Bonferroni-corrected *p*-value demonstrated that the difference was significant between group CC and H1 (*p* = 0.002), and the difference between group CC and H2 was significant (*p* = 0.0015), while there was no significant difference between group H1 and H2 (*p* = 0.14).

### Pearson correlation analysis

Pearson correlation analysis (Supplementary Figure S2) revealed several positive and negative correlation relationships. The digestibility of nutrients (DMD, OMD, CPD, EED, NDFD) has negative correlations with methane production and relative abundance of Spirochaetota, and positive correlations with VFA production, blood TAC and IgM, and the relative abundances of *Prevotella*, *Butyrivibrio*, *Acetitomaculum*, *Christensenellaceae R-7 group*, *Ruminococcus*, *Saccharofermentans,* and*Mogibacterium*.

### Reproductive performance

#### Changes in estrogen

**Fig. 3:**
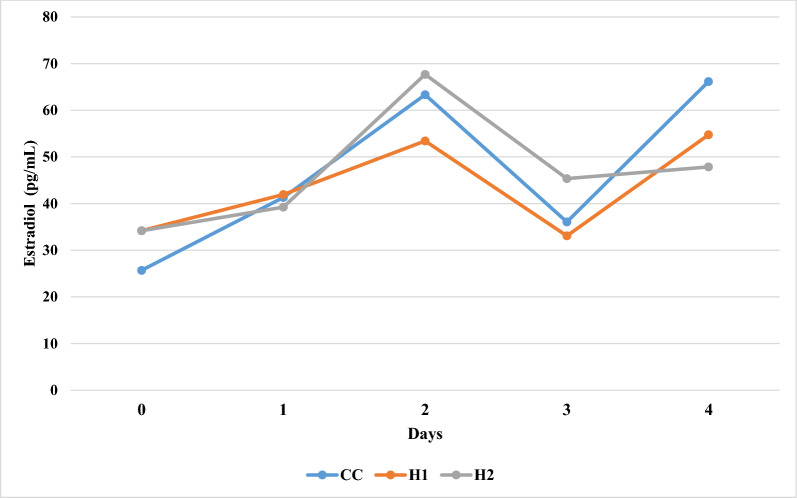
Changes in blood serum estrogen concentration (pg/ml) during four days after PGF2α injection in Shami goats. Blue line for control group (CC), orange line for goats supplemented with 1% herbal mixture (H1), and grey line for goats supplemented with 2% herbal mixture

The levels of estrogen were affected by the supplementation (*p* = 0.034) and collection time (*p* = 0.0001). Regarding the effect of collection time, groups CC and H1 showed their lower estrogen values at day-0, while higher values were observed at day-4 (Fig. 3). Moreover, group H2 showed a lower estrogen value at day-0 and a higher value at day-2. Regarding the supplementation effect, the estrogen levels were numerically higher (*p* > 0.05) at day-0 in supplemented groups (H1 = 34.2 and H2 = 34.18 pg/ml) compared to the control group (CC = 25.7 pg/ml) (*p *> 0.05). Group H1 showed lower estrogen levels at Day-2 (53.42) and Day-3 (33.08), while group H2 showed higher values at Day-2 (67.7) and Day-3 (45.36). At Day 4, group H2 showed the lowest estrogen value (47.86) and group CC showed the highest value (66.16).

#### Reproductive performance indices

The results showed that the conception and kidding rate were numerically higher in group H1 compared to group H2 and CC, without a significant difference (*p* > 0.05). Litter size was numerically higher in the control group (CC) without a significant difference (*p* > 0.05). The supplementation affected the sex ratio of the offspring whenever group H2 had a higher female-to-male ratio (*p* < 0.05).

## Discussion

Exploring the phytogenic compounds and their biological activities in different herbal plant mixtures has been of great interest in improving animal efficiency (Hassan et al. 2020; Rabee et al. 2024). The herbal mixture in the current study has a diverse range of bioactive compounds and a broad spectrum of biological actions as they manipulate the rumen microbial ecosystem and enhance the digestibility of nutrients, rumen fermentation, immunity, antioxidant capacity, and reproductive performance; therefore, it can be used as a feed additive. The herbal mixture used in the current study contains phenols, tannins, and flavonoids as well as different nutrients such as protein, fat, and minerals. Therefore, the inclusion of this phytogenic mixture in the goats’ diet with different levels modified the rumen bacterial community.

### Effect of the supplementation on the bacterial community

The supplementation enhanced observed ASVs and Chao indices; similar results were reported on buffalo and sheep fed phytogenic mixture (Patra et al. 2019; Hassan et al. 2020) or growing goats fed herbal mixture (Rabee et al. 2024). The dietary nutrients and antimicrobial activities of bioactive compounds explain their microbial fermentation-modifying properties (De la Cruz Gómez et al., 2024). Higher microbial diversity was associated with higher energy metabolism and forage digestion in goats (Belanche et al. 2023).

Phyla Bacteroidota and Firmicutes dominated the bacterial community, which agrees with goats and buffalo supplemented with different levels of herbal mixtures (Hassan et al. 2020; Rabee et al. 2024). Phylum Bacteroidota was classified mainly to the genera *Prevotella*, *Rikenellaceae RC9 gut group*, and unclassified families F082 and Bacteroidales BS11 gut group. Genus *Prevotella* was higher in supplemented groups; this genus is a key player in rumen fermentation as it degrades different substrates such as protein, peptides, hemicellulose, and produces propionate (Rabee et al. 2024, 2025a). The *Rikenellaceae RC9 gut group* was lower in group H2, which received a 2% herbal mixture. This genus was enriched in goats supplemented with 1% herbal mixture compared to the control group (Rabee et al. 2024). These findings indicate that this genus affords a specific concentration of phytochemicals. Similarly, this genus showed higher representation in cows supplemented with a lower dose of *Phyllanthus emblica,* which is rich in phytochemicals (Tilahun et al. 2024). Genus *Rikenellaceae RC9 gut group* ferments dietary fiber to produce acetic and propionic acids, succinate, which consume hydrogen from the rumen and decrease methane production (Andrade et al. 2022; Rabee et al. 2025a). The decline of this genus due to the supplementation of more than 1%, highlights this dose as a suitable dose to improve rumen fermentation.

Family F082 has a potential role in the degradation of soluble carbohydrates (Rabee et al. 2025a). The members of this family were lowere in the supplemented groups, indicating sensitivity to phytochemicals. This speculation is supported by the higher relative abundance of this family in tannin-extracted atriplex incubated in the rumen of camels (Rabee et al. 2023). Bacteroidales BS11 gut group revealed higher prevalence in the rumen of Yak fed a forage diet and degrades hemicellulose monomers such as xylose, fructose, mannose, and rhamnose, and produces acetate and butyrate (Liu et al. 2019). The decline in the relative abundance of this family due to supplementation indicates the sensitivity of this family to phytochemicals.

Within the phylum Firmicutes, the supplementation increased the relative abundance of *Butyrivibrio*, *Christensenellaceae R-7 group*, *Saccharofermentans*, *Ruminococcus*, and *Lachnospiraceae NK3A20 group*. *Butyrivibrio* and *Ruminococcus* have fibrolytic activities and produce different types of VFAs (Qi et al. 2024) and degrade different types of phytochemicals such as phenols and tannins (Rabee et al. 2023). *Christensenellaceae R-7 group*, which was higher in supplemented groups, is involved in fiber and protein fermentation and produces acetic and butyric acids; besides, it was more prevalent in efficient animals (Rabee et al. 2025a). Additionally, genus *Saccharofermentans* can degrade fiber and utilize glucose to produce acetate, succinate, and lactate; furthermore, it was enriched in the rumen of sheep supplemented with green husk of *Juglans regia* L., which is rich in polyphenols and flavonoids (Wei et al. 2024). *Lachnospiraceae NK3A20 group* is involved in carbohydrate metabolism and the production of acetic acid and butyric (Hou et al. 2025). Other genera that were higher in supplemented groups included *Acetitomaculum, Lachnospiraceae XPB1014 group*, *Syntrophococcus*, and *NK4A214 group*. Genus *Acetitomaculum* is an acetogenic bacteria that produce acetic acid using H_2_, which reduces the methane production and energy loss, which enhances feed efficiency (McLoughlin et al. 2023). The *Lachnospiraceae XPB1014 group* was positively correlated with the milk solids, milk protein, and milk fat (Liu et al. 2022). *Syntrophococcus* showed higher representation in the rumen of healthy dairy cows fed a high-forage diet and producing acetic acid (Wang S et al. 2023). *NK4A214 group* was enriched in the rumen of goats fed an herbal mixture (Rabee et al. 2024). This genus was associated with higher growth and lactation performance in ruminants, and produces propionate and butyrate (Wang D et al.^ 2023^; Chen et al. 2024).

Genus *Sphaerochaeta* within phylum Spirochaetota was decreased in the supplemented group; a similar finding was obtained in lambs supplemented with different types of condensed tannins (Salami et al. 2018). Genus *Fretibacterium* within phylum Synergistota was higher in goats fed 2% HM. This genus showed adaptability to different types of phytochemicals (Rabee et al. 2023) and has a role in lipid metabolism in the rumen (Yu et al. 2025).

### Effect of the supplementation level on the digestibility and rumen fermentation

The supplemented goats showed higher prevalence of bacterial genera that have important roles in fiber degradation, such as *Prevotella*, *Rikenellaceae RC9 gut group*, *Christensenellaceae R-7 group*, and *Saccharofermentans*. This finding explains the improvements in the digestibility and feed intake of nutrients in the supplemented groups. A similar finding was observed in dairy goats, cows, and buffalo supplemented with a polyherbal mixture (Mirzaei et al. 2012; Dey et al. 2021; Kholif et al. 2021) and goats supplemented with a herbal mixture (Rabee et al. 2024, 2025b). Furthermore, phytogenic compounds improved the microbial diversity and digestibility in sheep (Patra et al. 2019). On the other hand, herbal mixture provides micro-elements such as vitamins, hormones, and enzymes which support rumen microbiota and are required for efficient digestion, absorption, and metabolism (Mirzaei et al. 2012; Dey et al. 2021). Additionally, herbal mixtures improve digestion by improving the post-rumen digestive enzymes (Wang D et al. 2023). Higher feed intake in the supplemented groups indicates that the type of supplementation has no inhibitory impact on feed intake, while the decline in the feed intake in group H2 (fed 2% of HM) compared to H1 (fed 1% of HM) indicates that the dose of the supplementation affected the feed intake (Dey et al. 2021; Kholif et al. 2021). Furthermore, the oils in herbal plants improve the palatability and the digestibility of nutrients, which improves the feed intake (Kholif et al. 2021). However, increasing the herbal mixture supplementation level increases the concentration of phytogenic substances that might depress feed intake compared to lower doses (Kholif et al. 2021).

Rumen pH was not affected due to the supplementation, which agrees with the results of sheep fed phytogenic compounds (Patra et al. 2019). Neutral rumen pH is essential to keep the activities of starch and fiber-degrading bacteria (Kholif et al. 2021). The increment in total VFA, propionate, and acetate was also indicated in dairy cows fed different levels of phytogenic mixture (Kholif et al. 2021) and goats supplemented with phytochemicals (Rabee et al. 2024). Kholif et al. (2021) explained that improved VFA production is a result of improved rumen fermentation, and improved acetate is a result of higher fiber digestibility. These findings are supported by studies on goats (Rabee et al. 2024, 2025b), which indicated that phytochemicals enhanced fiber-degrading bacteria, digestibility of nutrients, and VFA concentration. The decline in methane due to the supplementation agrees with findings on buffalo supplemented with an herbal mixture (Dey et al. 2021). Lower methane contributes to improved animal efficiency as methane represents a loss of 2–12% of gross energy feed intake (Dey et al. 2021; Rabee et al. 2025a). The lower methane is associated with an increase in the propionic-producing bacteria, such as *Prevotella*, and rumen propionic acid production. The production of propionic acid absorbs the rumen hydrogen, which suppresses the methanogenesis (Dey et al. 2021; Rabee et al. 2024, 2025a).

### Effect of the supplementation level on blood parameters and immunity

The results demonstrated similar TG, TP, ALB, ALT, AST, and creatinine among experimental groups. Similar findings were obtained in dairy goats fed a herbal mixture (Rabee et al. 2025b; Wang D et al. 2023). These findings indicate normal physiology, health, and safety of the herbal supplementation (Dey et al. 2021; Kholif et al. 2021). Higher blood glucose in supplemented groups was previously reported in dairy cows and goats supplemented with a phytogenic mixture (Kholif et al. 2021; Rabee et al. 2025b). Higher blood glucose agrees with the improvement of nutrients’ digestibility (Dey et al. 2021; Kholif et al. 2021; Rabee et al. 2025b).

Lower blood urea in group H1 without affecting liver function (ALT and AST) agrees with the results on buffalo and cows (Dey et al. 2021; Kholif et al. 2021). This finding is attributed to the efficiency of protein utilization that occurs through inhibiting the protein degradation in the rumen by tannins or inhibiting the ammonia-producing bacteria by lipids in the herbal mixture (Dey et al. 2021). The similar liver functions (ALT and AST) among the goat groups indicate normal liver function and the safety of the supplementation (Kholif et al. 2021). Moreover, the decline in blood cholesterol was indicated in the cows fed different levels of phytogenic mixtures (Kholif et al. 2021) and goats fed herbal mixtures (Rabee et al. 2025b). Kholif et al. (2021) explained that phytogenic compounds inhibit enzymes involved in cholesterol synthesis.

Higher blood immunity (IgM) in supplemented groups was also indicated in dairy buffalo fed phytochemicals (Dey et al. 2021; Hashem et al. 2024) and goats supplemented with an herbal mixture (Rabee et al. 2025b; Wang D et al.^ 2023^). Phytochemicals such as flavonoids stimulate the production of lymphocytes, stimulate the immune system, and have anti-inflammatory activities (Hashem et al. 2024). Additionally, the improvement in immunity could be attributed to the improved feed utilization that stimulates the immune functions (Dey et al. 2021; Rabee et al. 2025b). The improvement in antioxidant capacity was previously noted in cows fed different levels of phytogenic mixture (Kholif et al. 2021), dairy goats supplemented with herbal mixture (Wang D et al.^ 2023^), and buffalo fed clover hay rich in flavonoids (Hashem et al. 2024). The improvement in TAC indicates improvement in animal health as phytogenic compounds improve the cellular antioxidant enzymes and scavenge reactive oxygen species that reduce injury to cells and tissue (Kholif et al. 2021).

### Reproductive performance

Higher estrogen in the supplemented groups was also indicated in buffalo fed *Trifolium alexandrinum* hay, which contains phytochemicals with estrogenic compounds (Hashem et al. 2024). The herbal mixture improves the reproduction performance through improving feed utilization, immunity, and antioxidant capacity, as well as regulating the reproductive hormones (Swelum et al. 2021; Wang D et al. 2023). Flavonoids increase the concentration of estrogen, which increases the follicular growth and number and diameters of follicles (Hashem et al. 2024).

Goat group H1 fed on 1% herbal mixture had higher conception and kidding rates, while higher herbal supplementation (2%) in group H2 decreased the conception and kidding rate compared to group H1. Similarly, buffalo fed on a diet with higher phytochemicals showed a lower conception rate (Hashem et al. 2024). Herbal mixture supplementation improved the reproductive performance parameters in Black Bengal goats (Singh et al. 2025) and dairy goats (Wang D et al. 2023). Furthermore, supplementing goats and cattle with yucca, which is rich in phenolic compounds, improved conception rates and kidding rates (Swelum et al. 2021), which supports the current results. However, no available information on the effect of supplementation level on reproduction performance. Higher conception and kidding rates suggest the compounds in the herbal mixtures have positive effects on the development of follicles, stimulate follicle maturation and ovulation, and ultimately increase estrus rate, embryo rate, and kidding rate (Wang D et al.^ 2023^). The negative effect of phytogenic substances is attributed to their hormone-like effects that lead to hormonal imbalance through elevated estrogen to progesterone, leading to silent heat, infertility, lower conception rate, and embryonic loss (Swelum et al. 2021; Hashem et al. 2024).

The effect of pre-conceptual maternal diet on the offspring sex ratio was previously reported (Rosenfeld et al., 2004). Goats supplemented with 2% herbal mixture (H2) had higher female offspring than other groups. The mode of action of maternal diet on sex differentiation still needs more explanation (Marei et al. 2018). Female mice supplemented with omega-6 polyunsaturated fatty acids gave birth to more females than males (Fountain et al. 2008). In contrast, cows supplemented with omega-6 polyunsaturated fatty acids gave birth to more males than females (Marei et al. 2018). Unfortunately, the fatty acids in the animal diets or the herbal mixture were not determined. However, analyses of fatty acids of herbal plants in the studied herbal mixture reported that these plants are rich in polyunsaturated fatty acids (PUFA) (Sulieman et al. 2008; Carvalho et al. 2011; Akbari et al. 2024). PUFAs represent the major portion of the fatty acids of follicular fluid and could affect oocyte cellular functions and developmental potential; furthermore, follicular PUFAs are affected by diet quality (Marei et al. 2018). The maternal hormonal profile at the time of conception affects offspring sex ratios (James 2006). Since some phytochemicals have phytoestrogenic effects due to structural similarity to mammalian estrogen, they bind with mammalian estrogen receptors (Hashem and Soltan 2016). Therefore, phytochemicals disrupt the hormonal balance, which affects the sex development and sex ratio of the offspring (Kim and Park 2012; Wang D et al. 2023).

Additionally, the herbal mixture has been reported to contain gallic acid, chlorogenic acid, caffeic acid, ellagic acid, and vanillin (Table 1). These compounds can break up the radical chain reaction by transforming free radicals into stable products since they are potent electron donors (Lobo et al. 2010). This process improves ratios of macrophages, leukocytes, and cytokines in the follicular fluid and are considered a major source of reactive oxygen species (ROS). ROS are involved in follicular growth by regulating angiogenesis and maturation by increasing the metabolic function of granulosa cells and ovarian steroid biosynthesis (Du et al. 2006), which may affect the follicles development and sex ratio of the offspring. Our results of estradiol 17-ß and total antioxidant capacity (Table 3; Fig. 3) and reproductive performance (Table 6) support this theory.

**Table 6:** Effect of herbal mixture supplementation level on the reproductive performance indices of goats

	CC	H1	H2	*p*-value
No. of does bred	12	12	12	ND
No. of does in oestrus	11	11	11	ND
No. of does concept	10	11	11	ND
No. of does kid	10	11	10	ND
Kidding rate	83.33	91.66	83.33	0.10
No. of kids born	22.00	22.00	17.00	ND
Litter size	2.20	2.00	1.70	0.51
Male	14.00	14.00	6.00	ND
Female	8.00	8.00	11.00	ND
Sex ratio (F/M)	57.14^b^	57.14^b^	183.33^a^	0.001
Single rate %	0	9.1 (1/11)	20 (2/10)	ND
Twins rate %	80 (8/10)	81.8 (9/11)	70 (7/10)	0.78
Triples rate %	20(2/10)	9.1(1/11)	10 (1/10)	0.71

Kidding rate = (number of dose lambed/number of doses exposed to bucks) *100;

Litter size born kids/kidded does; sex ratio (number of females/number of males) *100;

Single rate = number of dose single lambed/number of dose lamed) × 100;

Twins rate = number of dose twining lambed/number of dose lamed) × 100;

Triples rate = number of dose triples lambed/number of dose lamed) × 100

Therefore, the changes in sex ratio in group H2, due to higher phytochemical supplementation, are demonstrated and highlight the implications of the current herbal mixture on reproductive performance. Subsequently, herbal plants can be used to improve animal health as well as productive and reproductive performance (Kewan et al. 2021; Swelum et al. 2021).

## Conclusion

Supplementing the breeding goats with a phytogenic mixture modified rumen bacteria through enhancing the fiber-degrading bacteria, which improved the digestibility of nutrients, VFAs concentration, and feed intake, and decreased methane production. Additionally, the supplementation enhanced the immunity and antioxidant capacity, and reproduction performance indices such as the kidding rate. Thus, a herbal mixture consisting of ginger, garlic, artemisia, turmeric, fennel, and fenugreek can be supplied to breeding goats at 1% of DM intake. Future studies are recommended to apply this herbal mixture to pregnant and lactating animals, as well as growing animals.

## Supplementary Information

Supplementary Material 1.

Supplementary Material 2.

## Data Availability

The raw sequence reads are available at https://www.ncbi.nlm.nih.gov/sra/PRJNA1291028.

## Abbreviations

ASVs: Amplicon sequence variants
CFM: Concentrate feed mixture
DM: Dry matter
ELISA: Enzyme-linked immunosorbent assay
PERMANOVA: Bray–Curtis permutational multivariate analysis of variance
PCA: Principal component analysis
PCoA: Principal coordinate analysis
TAC: Total antioxidant capacity
VFA: Volatile fatty acids
