# Supplementary material for: Reproductive performance, digestibility, and rumen bacteria of goats fed two levels of phytogenic mixture
Source: AMB Express. 2025 Nov 3;15:163. doi: 10.1186/s13568-025-01961-y (PMC12583288; doi:10.1186/s13568-025-01961-y)
Supplement: Supplementary file 1 — Supplementary Material 1. [file 13568_2025_1961_MOESM1_ESM.pdf]

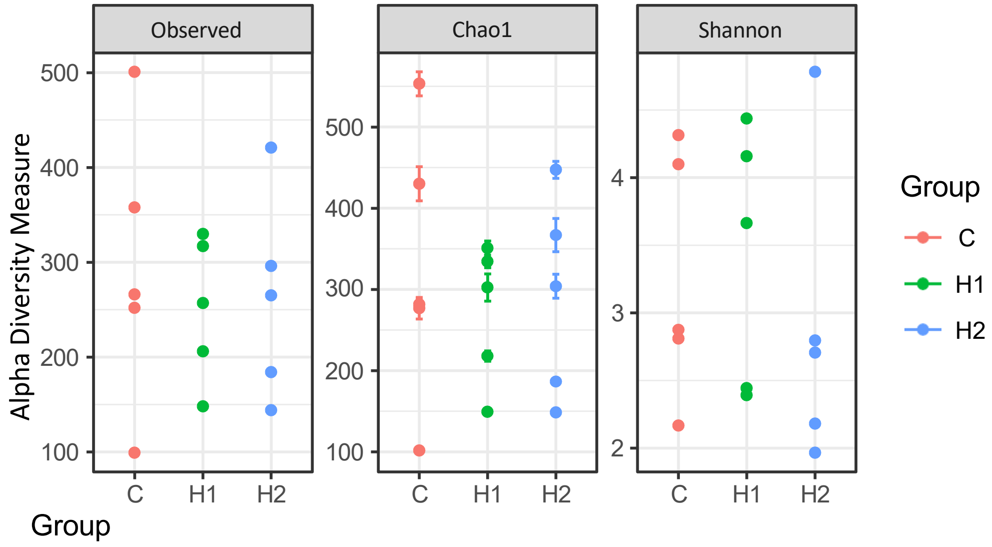

Supplementary Figure S1: Alpha diversity indices of bacterial community, including observed species, Chao1, and Shannon. The analyses were performed on three goat groups: red circles for the control group (CC), green circles for the goats supplemented with 1% herbal mixture (H1), and blue circles for the goats supplemented with 2% herbal mixture (H2).
