# Supplementary material for: Reproductive performance, digestibility, and rumen bacteria of goats fed two levels of phytogenic mixture
Source: AMB Express. 2025 Nov 3;15:163. doi: 10.1186/s13568-025-01961-y (PMC12583288; doi:10.1186/s13568-025-01961-y)
Supplement: Supplementary file 2 — Supplementary Material 2. [file 13568_2025_1961_MOESM2_ESM.pdf]

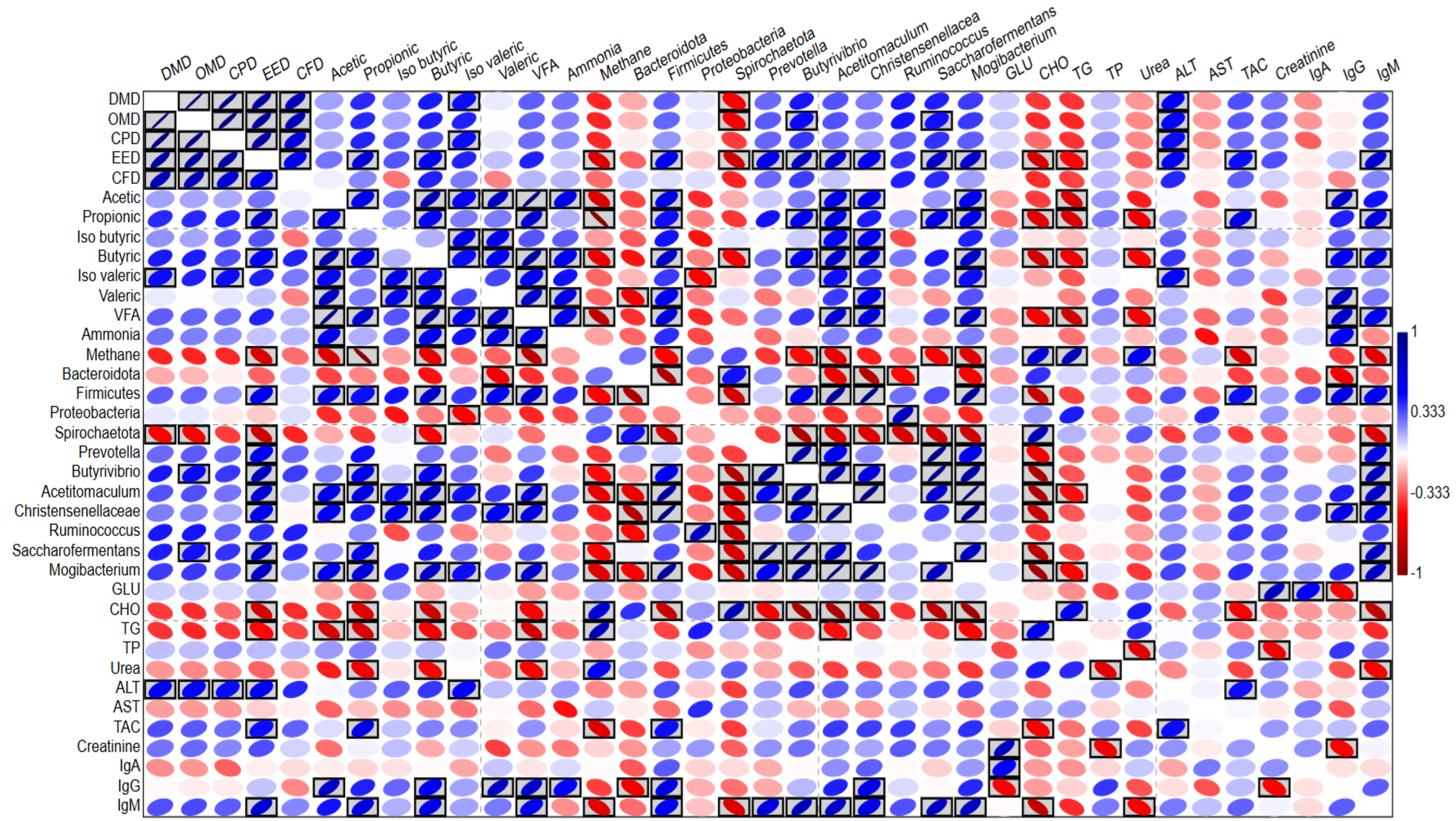

Supplementary Figure S2: Heatmap based on Pearson correlation. The correlation was conducted using the data of digestibility of nutrients, rumen fermentation parameters and bacteria, and blood metabolites of goats supplemented with different levels of herbal mixture. The black boxed ellipses indicate to significant correlations at $P < 0.05$
